# The Longitudinal Associations of Mothers’ and Fathers’ Parenting Styles with Preschool Children’s Social Problem-Solving Skills: The Moderating Role of Primary-Caregiver Status

**DOI:** 10.3390/bs16081447

**Published:** 2026-08-21

**Authors:** Keman Yuan, Jiaxin Sun, Yufang Bian

**Affiliations:** 1 Shanghai Institute of Early Childhood Education, Shanghai Normal University, Shanghai 200233, China; kmyuan@shnu.edu.cn (K.Y.); 1000588774@smail.shnu.edu.cn (J.S.); 2 Collaborative Innovation Center of Assessment Toward Basic Education Quality, Beijing Normal University, Beijing 100875, China

**Keywords:** parenting styles, social problem-solving skills, primary-caregiver status, preschool children

## Abstract

The bioecological model emphasizes parent–child interactions as proximal processes associated with children’s development over time, whereas family systems theory highlights the distinct yet interdependent roles of mothers and fathers. However, few longitudinal studies have simultaneously examined the associations of mothers’ and fathers’ parenting styles with preschool children’s social problem-solving skills. This study recruited 275 co-residing mother–father Chinese families and employed a three-wave longitudinal design to investigate the longitudinal associations between fathers’ and mothers’ parenting styles and preschool children’s social problem-solving skills, while considering primary-caregiver status. After accounting for prior levels, mothers’ authoritative parenting at Time 2 was positively associated with children’s social problem-solving skills at Time 3, whereas mothers’ authoritarian parenting at Time 1 was negatively associated with social problem-solving skills at Time 2. Fathers’ authoritative parenting showed no significant longitudinal association. However, the interaction between authoritarian parenting at Time 1 and whether the father was identified as the primary caregiver was significantly associated with children’s social problem-solving skills at Time 2. These findings indicate limited and interval-specific longitudinal associations between parenting styles and children’s social problem-solving skills and the possible relevance of primary-caregiver status.

## 1. Introduction

Social problem-solving skills encompass cognitive, emotional, and behavioral processes. Through these processes, children identify social problems from relevant interpersonal cues, understand their possible causes, generate and evaluate alternative solutions, and select appropriate strategies (Ayık et al., 2025). These skills integrate emotional understanding, cognitive analysis, and interpersonal strategies to address specific social problems, rather than merely recognizing others’ emotions or displaying appropriate social behavior. As one of the five core dimensions of social-emotional competence in preschool children, social problem-solving skills are related to maintaining positive social interaction and social-emotional adaptation (Chang et al., 2020). The bioecological model of human development (Bronfenbrenner & Morris, 2006) places particular emphasis on proximal processes. It posits that individuals engage in complex interactions with the people, objects, and symbols in their immediate environment, and these interactions are fundamental to development over time. Within the family microsystem, repeated parent–child interactions constitute important proximal processes through which parenting styles may be associated with preschool children’s social development (Darling & Steinberg, 1993; Gallagher, 2002). Moreover, family systems theory conceptualizes the family as an organized and interdependent whole in which both mothers and fathers contribute to children’s development (Cox & Paley, 1997). Although mothers’ and fathers’ parenting may be differentially associated with developmental outcomes, their roles are interconnected and should not be understood as operating independently of one another (Martin et al., 2007). Previous studies examining the relationship between parenting styles and general social-emotional competence have primarily used cross-sectional designs and have rarely distinguished between the roles of mothers and fathers. The present study employs a three-wave longitudinal design to investigate the associations between authoritative and authoritarian parenting styles and preschool children’s social problem-solving skills. The study also examines whether primary-caregiver status moderates these associations separately for mothers and fathers. The findings are expected to clarify the longitudinal associations between mothers’ and fathers’ parenting styles and preschool children’s social problem-solving skills. They may also advance understanding of the potential moderating role of primary-caregiver status.

### 1.1. Parenting Styles and Social Problem Solving Skills of Preschool Children

Parenting style refers to the emotional attitudes shown by parents during the process of communication and interaction with their children and the emotional climate established through these attitudes. It is a combination of a series of parenting attitudes and practices of parents towards their children in daily life (Darling & Steinberg, 1993), and is commonly categorized as authoritative, authoritarian, permissive, and neglectful (Baumrind, 1989; Maccoby & Martin, 1983). Cross-cultural research has found that Chinese parents more often adopt authoritative and authoritarian parenting styles (e.g., F. M. Chen & Luster, 2002). This tendency can be attributed to traditional Chinese cultural values, which prioritize parental authority and children’s filial piety. In the process of raising their children, Chinese parents not only provide love, attention, and support to their children, but also discipline their children through guidance and long-term supervision (Chao, 2000). Accordingly, this study mainly focuses on the associations of authoritative and authoritarian parenting styles with the social problem-solving skills of preschool children.

Parents who adopt an authoritative parenting style tend to be responsive to their children’s needs, provide warmth and support, respect their autonomy, and regulate their behavior through reasoning. Robinson et al. (1995) conceptualized the authoritative parenting style as comprising three practices: warmth, reasoning, and autonomy support. Parental warmth, which involves timely and empathetic responses tailored to children’s situational needs during interactions, has been associated with the development of children’s social-emotional competence (Fung, 2022; Orbán et al., 2025). Through such responses, children are able to acquire a sense of security when interacting with the outside world. Meanwhile, parents can offer personalized guidance, which is likely to promote children’s early development of language skills, such as the ability to express and understand emotion-related vocabulary (Shipkova et al., 2025). Reasoning involves guiding and regulating children’s behavior through logical explanations. When children encounter emotional or social challenges, authoritative parents tend to address these difficulties collaboratively and discuss strategies for managing emotions across contexts, which may enhance children’s emotion-regulation skills (Shipman & Zeman, 2001) as well as their ability to solve problems through friendly negotiation with peers. Autonomy support emphasizes children’s independence and self-efficacy and has been associated with preschoolers’ peer communication skills (Martinez, 1987). These capacities may be relevant to the use of constructive strategies during social conflicts. However, most previous studies have used cross-sectional designs to examine the associations between authoritative parenting style and outcomes such as social skills and emotional competence (e.g., Huang et al., 2024). The association between the authoritative parenting style and children’s social problem-solving skills therefore remains insufficiently understood and warrants further investigation.

In contrast, parents who adopt an authoritarian parenting style place a strong emphasis on obedience, expecting children to respect authority and follow instructions. They also tend to regulate children’s behavior through coercive means. Robinson et al. (1995) identified three practices of authoritarian parenting style: verbal hostility, corporal punishment, and non-reasoning punitive strategies. Authoritarian parents are generally less responsive to their children’s emotional needs. Lower parental responsiveness to children’s emotions has been associated with lower empathy, weaker emotion-regulation skills, and poorer social competence (Anthony et al., 2005; Goagoses et al., 2023; Smith et al., 2001). D. Li et al. (2024) reported that authoritarian parenting style was associated with poorer peer interactions and more negative social relationship patterns. Furthermore, social learning theory (Bandura & Walters, 1977) suggests that children learn by observing and imitating their parents’ behavior. Thus, children exposed to coercive or aggressive parenting practices may be more likely to adopt similarly aggressive strategies when addressing social problems. However, Chinese culture traditionally emphasizes intergenerational hierarchy, parental authority, and strict discipline. Accordingly, Chinese parents may adopt highly directive and controlling parenting practices (Chao, 1994). Some research has linked the authoritarian parenting style to positive developmental outcomes among children in Chinese American families (X. Chen et al., 2001), whereas Ren and Edwards (2015) found that it did not significantly predict preschool children’s social skills. These mixed findings highlight the need to further examine the association between authoritarian parenting style and preschool children’s social problem-solving skills within the Chinese cultural context.

### 1.2. The Difference Between Mothers and Fathers

Previous research on parenting styles and child development has largely focused on mothers. Other studies have combined mothers’ and fathers’ parenting into a single measure, often by averaging their scores. Consequently, fathers’ contributions to child development have received comparatively limited attention, and few studies have examined differences between mothers and fathers. This imbalance may partly reflect traditional divisions of parental caregiving responsibilities. Fathers were often expected to provide financial support and were less involved in daily childcare (Eagly et al., 2000). However, fathers’ involvement in parenting has increased alongside broader social changes (Cabrera et al., 2000).

Mothers’ and fathers’ parenting may differ in both their typical levels and their associations with child outcomes. Regarding parenting styles, earlier studies suggested that mothers were more likely to adopt an authoritative style, whereas fathers were more likely to adopt an authoritarian style. These differences may partly reflect traditional Chinese cultural expectations embodied in the notion of “strict fathers and kind mothers.” Within this framework, fathers are assigned greater authority within the family. Fathers have traditionally been expected to establish rules, regulate children’s behavior, and oversee their academic and personal development, whereas mothers have typically been expected to provide emotional warmth, manage children’s daily care, and meet their basic needs (Shek, 2000).

Previous research has reported different patterns of association between mothers’ and fathers’ parenting and certain aspects of children’s development (Day & Padilla-Walker, 2009). For example, Xing et al. (2018) found that maternal corporal punishment was significantly associated with greater executive dysfunction in children, whereas the corresponding association for paternal corporal punishment was non-significant. Xu et al. (2018) suggested that comparatively weaker paternal associations might be related to fathers’ lower levels of involvement and shorter amounts of time spent with their children. From the perspective of social capital theory, the time parents spend with their children constitutes an important form of family social capital (Alcaraz et al., 2020). Greater father involvement may create more opportunities for parent–child interaction and may be associated with more pronounced links between parenting and child development.

However, fathers’ caregiving roles have evolved alongside broader social changes. As women’s participation in the workforce has increased, fathers have assumed greater responsibilities for household tasks, childcare, and parenting (Zhang, 2013). It is therefore important to examine mothers’ and fathers’ parenting styles separately and compare their associations with children’s social problem-solving skills.

### 1.3. The Present Study

This study employed a three-wave longitudinal dyadic design to systematically examine the associations of mothers’ and fathers’ authoritative and authoritarian parenting styles with preschool children’s social problem-solving skills. It further investigated whether primary-caregiver status moderated these associations. Accordingly, the following hypotheses were proposed:

**H1.**  *After accounting for prior levels of children’s social problem-solving skills, mothers’ and fathers’ authoritative parenting would each be positively associated with children’s subsequent social problem-solving skills.*

**H2.**  *After accounting for prior levels of children’s social problem-solving skills, mothers’ and fathers’ authoritarian parenting would each be negatively associated with children’s subsequent social problem-solving skills.*

**H3.**  *Primary-caregiver status would moderate the longitudinal associations between parenting styles and children’s social problem-solving skills, such that these associations would be stronger when the corresponding parent was identified as the primary caregiver.*

## 2. Materials and Methods

### 2.1. Participants

Using cluster sampling, this study recruited the parents of 418 preschool children (*M*_age_ = 4.46, *SD* = 0.74; 53.1% boys) from eight kindergartens in Beijing, China, in early June 2023 (the end of the spring semester, Time 1). Two follow-up questionnaire surveys were administered to these families in September (the beginning of the fall semester, Time 2) and December (the end of the fall semester, Time 3). Due to parental voluntary withdrawal, children transferring to other kindergartens, and other personal reasons, 305 families participated at Time 2, and the final sample at Time 3 consisted of 282 families, corresponding to an attrition rate of 32.54%. No significant differences were observed between the Time 3 and Time 1 samples in fathers’ parenting styles, mothers’ parenting styles, children’s social problem-solving skills, children’s age, or subjective family socioeconomic status (SES), with *t*-values ranging from 0.02 to 1.94, *p*s > 0.05, Cohen’s *d*s < 0.20; and no significant group differences were found in children’s gender, *χ*^2^_(1)_ = 0.11, *p* > 0.05, or only child status, *χ*^2^_(1)_ = 2.28, *p* > 0.05. To ensure response quality, questionnaires completed in less than 5 min or exhibiting patterned response patterns were excluded. The final effective sample consisted of 275 parent pairs, with mothers’ mean age at Time 1 being 36.10 years (*SD* = 4.28) and fathers’ mean age at Time 1 being 37.78 years (*SD* = 5.23). Mothers were classified as the primary caregivers in 237 families (86.2%; children’s mean age = 4.41 years, *SD* = 0.78; 53.2% boys; 62.4% only children), whereas fathers were classified as the primary caregivers in 38 families (13.8%; children’s mean age = 4.79 years, *SD* = 1.20; 52.6% boys; 76.3% only children). The two groups did not differ significantly in subjective family SES, *t*_(273)_ = 0.30, *p* > 0.05.

### 2.2. Procedures

Ethical approval was obtained from the Academic Ethics Committee of the Collaborative Innovation Center of Assessment toward Basic Education Quality at Beijing Normal University. After obtaining approval from the kindergarten and teachers, researchers introduced the study to parents via posters and distributed the online questionnaire link through teachers, inviting both fathers and mothers to participate via Wenjuanxing (Changsha Ranxing Information Technology Co., Ltd., Changsha, China). At each wave, mothers and fathers completed separate online questionnaires and independently provided informed consent for their participation. The first page of the questionnaire provided informed consent, outlining the study’s purpose and data confidentiality. Participation was voluntary; parents could click “Agree” to begin or exit the survey at any time. The procedures were consistent across all three time points.

### 2.3. Measures

#### 2.3.1. Parenting Styles and Dimensions Questionnaire

Parenting styles were assessed using the Parenting Styles and Dimensions Questionnaire (Robinson et al., 1995), which consisted of three subscales: authoritative, authoritarian, and permissive. The authoritative parenting style consists of 15 items across three dimensions: warmth (7 items), reasoning (4 items), and autonomy support (4 items), with sample items such as “gives the child reasons why rules should be obeyed”. The authoritarian parenting style also comprises three dimensions: corporal punishment (5 items), verbal hostility (3 items), and non-reasoning (3 items), exemplified by an item like “punishes by taking privileges away with little explanation”. Both mothers and fathers completed the questionnaire separately, using a 5-point Likert scale (1= never to 5 = always), with higher average scores indicating stronger characteristics of the respective parenting style. The Chinese version of this questionnaire has been widely used in Chinese cultural contexts (e.g., Lin et al., 2022) and showed high reliability in this study, with Cronbach’s α coefficients for the subscales ranging from 0.80 to 0.88 for mothers and 0.77 to 0.89 for fathers across all three time points.

#### 2.3.2. Preschool Children’s Social and Emotional Competence Questionnaire

The Preschool Children’s Social and Emotional Competence Questionnaire, developed by Yuan (2023), was used to assess six dimensions of preschoolers’ social-emotional competence: self-awareness and expression, emotion and behavior regulation, other-awareness, social initiative, awareness of rules and group consciousness, and social problem-solving skills (Appendix A for details). The questionnaire comprises 25 items, including three items specifically measuring social problem-solving skills (e.g., “After having a conflict with a friend, the child will make friendly gestures, such as giving small gifts”). The social problem-solving skills subscale was completed by the primary caregiver, who was the mother in 237 families (86.2%) and the father in 38 families (13.8%). Each item was rated on a 5-point scale (1 = Not true at all to 5 = Definitely true). Scale scores were calculated as the mean of all items, with higher scores indicating greater social problem-solving skills. This questionnaire has been validated through large-scale administration in the context of Chinese culture, S-B*χ*^2^ =1545.297, *df* = 260, CFI = 0.922, TLI = 0.910, RMSEA = 0.047, 95% confidence interval [0.044, 0.049], SRMR = 0.044. The Cronbach’s α coefficient for the social problem-solving skills subscales was 0.71 at Time 1, 0.76 at Time 2, and 0.75 at Time 3.

#### 2.3.3. Demographic Information

Demographic data for parents and children were collected via a brief questionnaire at Time 1. Parents reported their age and whether they were the primary caregiver. Specifically, primary caregiver status was assessed by asking both parents independently, “In your family, which parent spends relatively more time on the child’s daily care, including feeding, education, and emotional support?” The response options were “mother” and “father.” Mothers’ and fathers’ classifications showed high agreement (97.1% raw agreement; Cohen’s *κ* = 0.89). Their responses were discrepant in eight families; for these families, primary caregiver status was determined by comparing the parents’ separately reported average daily caregiving time. Additionally, primary caregivers reported the child’s gender, age, and only child status, whereas both parents rated subjective family SES on a 10-point scale (Adler et al., 2000). Their ratings were averaged, with higher mean scores indicating higher perceived family educational attainment, occupational status, and income.

### 2.4. Analytic Strategy

Data for this study were managed and analyzed using SPSS Statistics 21.0 and Mplus 8.0. First, descriptive statistics were calculated for parenting styles and preschool children’s social problem-solving skills. Second, longitudinal measurement invariance was tested for mothers’ and fathers’ parenting styles and children’s social problem-solving skills. Third, cross-lagged panel models for authoritative and authoritarian parenting styles were constructed separately. Child gender, age, only child status, and family SES were entered as control variables, with robust maximum likelihood estimation and Full Information Maximum Likelihood used to handle missing data. To examine potential omitted-variable bias arising from modeling authoritative and authoritarian parenting separately, sensitivity analyses were conducted in which each focal parenting dimension was examined while controlling for the alternative parenting dimension at the preceding time point. Full results are reported in Appendix B.

Finally, the latent moderated structural equations (LMS) approach (Klein & Moosbrugger, 2000) was employed to examine whether primary caregiver status moderates the association between parenting styles and children’s social problem-solving skills. To test this moderation effect, a structural equation model excluding the latent interaction term was first estimated as the baseline model (Model 0). The latent interaction term was then included in Model 1. Because Mplus does not report conventional fit indices for LMS models, the relative fit of the two nested models was evaluated using the Akaike information criterion (AIC) and a likelihood ratio test (Fang & Wen, 2018). A lower AIC value for Model 1 indicated that adding the latent interaction term did not worsen the model’s relative fit after accounting for the additional complexity, and a statistically significant *D* indicated that adding the latent interaction term significantly improved model fit. The likelihood ratio statistic, *D*, was calculated using Equation (1), where *LL*_0_ and *LL*_1_ denote the log-likelihood values of Models 0 and 1, respectively. The statistical significance of the latent product term was subsequently evaluated using its estimated coefficient and 95% confidence interval; an interval excluding zero indicated a statistically significant interaction coefficient (Hayes, 2013). When a significant interaction effect was detected, an interaction simple-slope analysis was conducted to facilitate its interpretation. Additionally, to evaluate the statistical power of the focal moderation effect, a parameter-based Monte Carlo simulation was conducted in Mplus using 1000 replications.

*D* = −2 × (*LL*_0_ − *LL*_1_) (1)

### 2.5. Common Method Bias Test

Since some parenting and child-outcome measures were completed by the same respondent, Harman’s single-factor test was conducted separately for the mother- and father-reporter groups at each wave to assess potential common method bias (Harman, 1976; Podsakoff et al., 2003). Among families in which mothers were identified as the primary caregivers, the first principal factor accounted for 29.99%, 31.10%, and 31.49% of the total variance at Times 1, 2, and 3, respectively. Among families in which fathers were identified as the primary caregivers, the corresponding percentages were 28.64%, 36.93%, and 37.92%. Although all values were below 40%, these results indicate only that no single dominant factor was detected. However, this analysis cannot exclude the possibility of common-source bias (Podsakoff et al., 2003).

## 3. Results

### 3.1. Preliminary Analyses

#### 3.1.1. Differences Between Mothers’ and Fathers’ Parenting Styles

Table 1 presents the means and standard deviations of mothers’ and fathers’ authoritative and authoritarian parenting styles. Paired-samples *t* tests indicated that mothers reported significantly higher levels of authoritative parenting than fathers at all three time points. However, mothers’ and fathers’ authoritarian parenting scores did not differ significantly at any time point.

#### 3.1.2. Correlations Between Parenting Styles and Children’s Social Problem Solving Skills

Table 2 presents the correlations among mothers’ and fathers’ parenting styles, children’s social problem-solving skills, and demographic variables across the three time points. Mothers’ authoritative parenting style was significantly and positively correlated with children’s social problem-solving skills both concurrently and across time (*r*s = 0.23~0.38). In contrast, most correlations involving mothers’ authoritarian parenting style were non-significant. Fathers’ authoritative parenting style was significantly and positively correlated with children’s social problem-solving skills at the same time points (*r*s = 0.13~0.16), whereas its cross-time correlations were either significantly positive or non-significant Fathers’ authoritarian parenting style was not significantly correlated with children’s social problem-solving skills, either concurrently or across time.

### 3.2. The Cross-Lagged Analysis of Parenting Styles and Children’s Social Problem Solving Skills

To examine the longitudinal associations between mothers’ and fathers’ authoritative and authoritarian parenting styles and preschool children’s social problem-solving skills, cross-lagged panel models were constructed using paired parent data. Before estimating the cross-lagged models, longitudinal measurement invariance was tested for authoritative parenting, authoritarian parenting, and children’s social problem-solving skills. Following Cheung and Rensvold (2002), an absolute change in CFI smaller than 0.01 was used as the criterion for evaluating measurement invariance, and changes in RMSEA and SRMR were also reported and considered as supplementary evidence. As shown in Table 3, full scalar invariance was supported for fathers’ authoritative parenting and mothers’ authoritative and authoritarian parenting. Partial scalar invariance was supported for fathers’ authoritarian parenting and children’s social problem-solving skills after selected intercept constraints were released. Partial scalar invariance was considered sufficient because at least two indicators for each construct retained invariant factor loadings and intercepts across the three waves, thereby providing adequate anchors for longitudinal comparisons (Vandenberg & Lance, 2000). In all accepted partial-invariance models, |ΔCFI| remained below 0.01. Although |ΔRMSEA| exceeded 0.015 and |ΔSRMR| exceeded 0.03 in some comparisons, ΔCFI was the prespecified primary decision criterion, whereas changes in RMSEA and SRMR were treated as supplementary evidence (F. F. Chen, 2007). Thus, the overall pattern of fit-index changes was interpreted as generally supporting longitudinal measurement invariance, although the inconsistent RMSEA results warrant some caution.

Further, separate cross-lagged models were constructed to examine the associations of authoritative and authoritarian parenting styles with children’s social problem-solving skills. Both cross-lagged panel models demonstrated acceptable fit. The authoritative parenting model yielded the following fit indices: S-B*χ*^2^ = 309.386, *df* = 245, CFI = 0.981, TLI = 0.976, RMSEA = 0.031, 90% CI [0.019, 0.041], and SRMR = 0.060. The authoritarian parenting model yielded S-B*χ*^2^ = 400.329, *df* = 245, CFI = 0.956, TLI = 0.944, RMSEA = 0.048, 90% CI [0.039, 0.056], and SRMR = 0.081.

**Figure 1:**
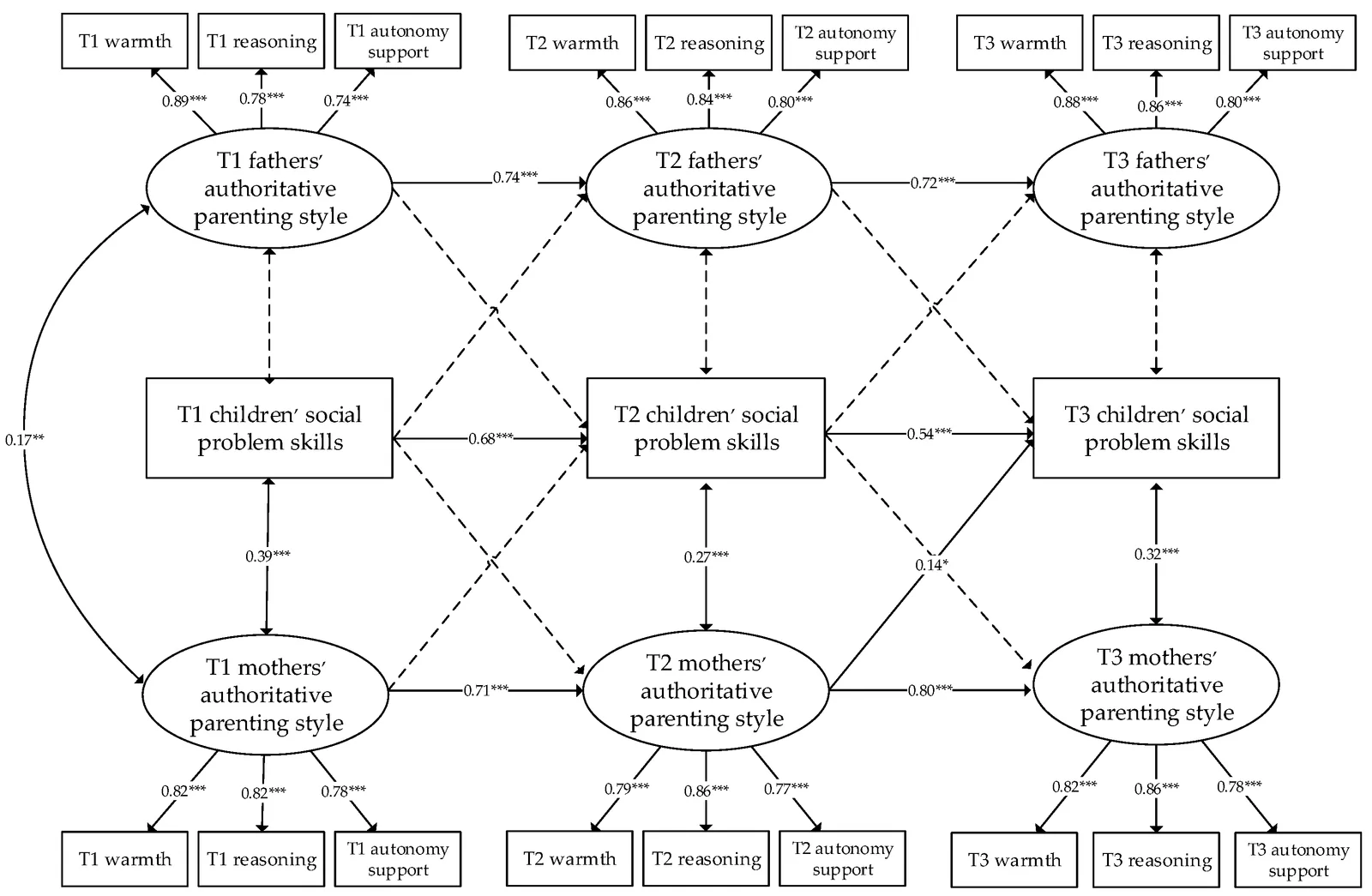
Cross-lagged model of mothers’ and fathers’ authoritative parenting styles and children’s social problem-solving skills. Note. Solid lines indicate statistically significant paths, whereas dashed lines indicate non-significant paths. Covariate paths and residual correlations are not displayed for visual clarity; * *p* < 0.05, ** *p* < 0.01, *** *p* < 0.001.

Figure 1 presents the cross-lagged model of the authoritative parenting style and children’s social problem-solving skills. Mothers’ authoritative parenting style was significantly and positively correlated with children’s social problem-solving skills at each time point (*β*s = 0.27~0.39), whereas the corresponding concurrent associations for fathers’ authoritative parenting style were non-significant. Significant autoregressive effects were observed for mothers’ and fathers’ authoritative parenting styles and children’s social problem-solving skills across adjacent time points (*β*s = 0.54~0.80), indicating substantial temporal stability in these constructs. Regarding the cross-lagged effects, mothers’ authoritative parenting style at Time 2 was significantly and positively associated with children’s social problem-solving skills at Time 3 after accounting for their prior skill levels (*β* = 0.14). Fathers’ authoritative parenting style showed no significant longitudinal association with children’s subsequent social problem-solving skills.

**Figure 2:**
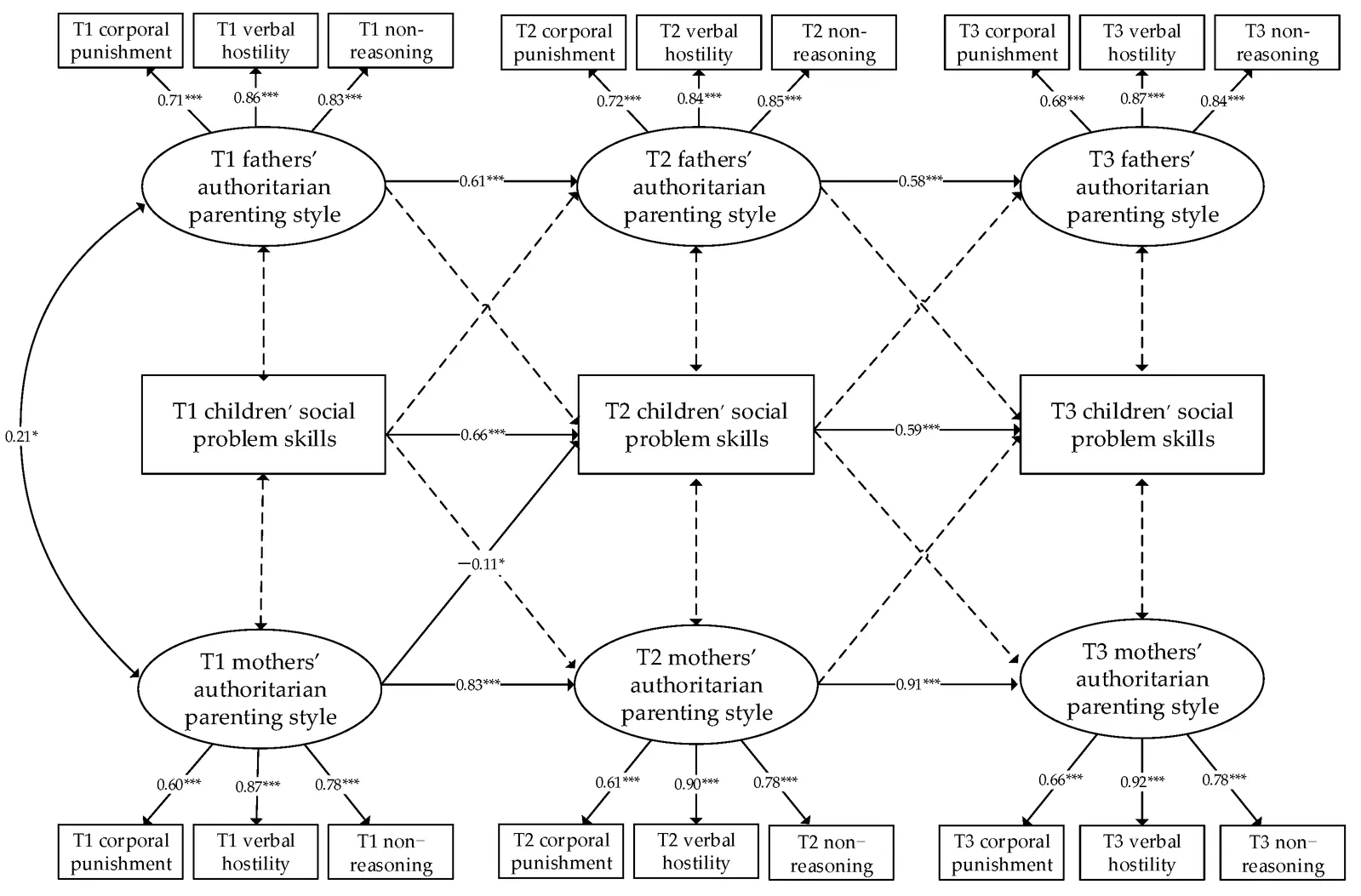
Cross-lagged model of mothers’ and fathers’ authoritarian parenting styles and children’s social problem-solving skills. Note. Solid lines indicate statistically significant paths, whereas dashed lines indicate non-significant paths. Covariate paths and residual correlations are not displayed for visual clarity; * *p* < 0.05, *** *p* < 0.001.

Figure 2 presents the cross-lagged model of parents’ authoritarian parenting style and children’s social problem-solving skills. Neither mothers’ nor fathers’ authoritarian parenting style was significantly correlated with children’s social problem-solving skills at any time point. Significant autoregressive effects were observed for mothers’ and fathers’ authoritarian parenting and children’s social problem-solving skills across adjacent time points (*β*s = 0.58~0.91), indicating temporal stability in these constructs. Regarding the cross-lagged effects, mothers’ authoritarian parenting at Time 1 was significantly and negatively associated with children’s social problem-solving skills at Time 2 after accounting for their prior skill levels (*β* = −0.11). Fathers’ authoritarian parenting showed no significant overall longitudinal association with children’s social problem-solving skills at the subsequent time point.

### 3.3. The Moderating Role of Primary-Caregiver Status

The primary-caregiver status was dummy coded (0 = mother as primary caregiver, 1 = father as primary caregiver), and the LMS analysis indicated that primary-caregiver status did not significantly moderate the longitudinal associations of fathers’ authoritative parenting or mothers’ authoritative or authoritarian parenting with preschool children’s social problem-solving skills. Although the AIC values decreased after the two interaction terms were added to each of these models, neither of the likelihood-ratio tests was statistically significant. Specifically, adding the two interaction terms did not significantly improve model fit for paternal authoritative parenting, *D*_(2)_ = 2.01, *p* = 0.366; maternal authoritative parenting, *D*_(2)_ = 4.12, *p* = 0.128; or maternal authoritarian parenting, *D*_(2)_ = 2.86, *p* = 0.239.

For fathers’ authoritarian parenting, the interaction between fathers’ authoritarian parenting at Time 1 and whether the father was identified as the primary caregiver was significant in relation to children’s social problem-solving skills at Time 2, *B* = −0.25, *SE* = 0.12, 95% CI [−0.481, −0.020], *p* = 0.03. In contrast, the interaction between fathers’ authoritarian parenting at Time 2 and fathers’ primary-caregiver status was non-significant in relation to children’s social problem-solving skills at Time 3, *B* = −0.03, *SE* = 0.14, 95% CI [−0.297, 0.248], *p* = 0.859. Although the AIC decreased slightly from 12,576.46 to 12,575.84 after the two interaction terms were added, the joint likelihood-ratio test for the two interaction terms did not indicate a significant improvement in model fit, *D*_(2)_ = 4.62, *p* = 0.099. The simple-slope results for the Time 1–Time 2 association are presented in Table 4. Given the inconsistency between the significant Wald test for the Time 1 interaction and the non-significant joint likelihood-ratio test, this moderation pattern was interpreted cautiously. The 1000-replication Monte Carlo simulation yielded an estimated power of 0.47 for the significant interaction between fathers’ authoritarian parenting at Time 1 and primary-caregiver status. Therefore, the result was interpreted as an exploratory and inconclusive interaction signal rather than evidence of confirmed moderation.

## 4. Discussion

### 4.1. The Longitudinal Association Between Parenting Styles and Social Problem Solving Skills of Preschool Children

This study employed a three-wave longitudinal design to examine the associations between mothers’ and fathers’ parenting styles and children’s social problem-solving skills in a Chinese sample. After accounting for prior levels of children’s social problem-solving skills, mothers’ authoritative parenting style at Time 2 was positively associated with children’s social problem-solving skills at Time 3, whereas mothers’ authoritarian parenting style at Time 1 was negatively associated with these skills at Time 2. It should be noted that these associations were interval-specific and were not replicated across both longitudinal intervals. For fathers, an exploratory interaction signal involving authoritarian parenting at Time 1 and primary-caregiver status was observed for children’s social problem-solving skills at Time 2. This pattern was interval-specific and was not observed from Time 2 to Time 3. Given this lack of replication across intervals and the limited statistical power of the moderation analysis, the finding should be interpreted cautiously. These findings extend previous cross-sectional research by identifying time-interval-specific longitudinal associations. They also suggest that the role of primary-caregiver status in fathers’ parenting warrants further investigation.

Mothers’ authoritative parenting style at Time 2 was positively associated with preschool children’s social problem-solving skills at Time 3. One possible explanation is that mothers who adopt an authoritative parenting style are generally warm, sensitive, and responsive to children’s needs. They also tend to regulate children’s behavior through reasoning rather than harsh punishment. Such a parenting style has been associated with secure mother–child attachment (Ren et al., 2019). According to attachment theory, parent–child attachment shapes children’s internal working models of themselves and others (Bowlby, 1973). Children with secure attachment are more likely to view interpersonal relationships as safe and supportive, which may help them approach peer conflicts in more constructive ways. Consistent with this view, Laible (2007) found that secure attachment was associated with children’s emotional understanding and positive emotional expression. Thus, when children encounter social problems, secure attachment may help them maintain emotional stability, understand peers’ emotional states, and use more empathic and constructive strategies to resolve conflicts. However, because attachment was not measured in this study, this post hoc interpretation requires direct examination in future research.

In addition, authoritative mothers typically monitor children’s behavior and set clear rules while also using supportive strategies, such as explanation and reasoning, when children behave inappropriately. These practices may be associated with children’s prosocial behavior (Laible, 2007) and may support cooperative and constructive responses to peer problems (Daglar et al., 2011). From the perspective of social learning theory, children acquire behavioral strategies by observing and imitating significant others (Bandura & Walters, 1977). Therefore, children may internalize mothers’ reasoning-based interaction patterns and apply similar strategies when solving social problems with peers. Together, these mechanisms may explain why mothers’ authoritative parenting was associated with the development of children’s social problem-solving skills. Although this possible pathway may help account for the observed interval-specific association, it remains a tentative interpretation because observational learning and strategy internalization were not directly measured.

A separate interval-specific finding was that mothers’ authoritarian parenting at Time 1 was negatively associated with children’s social problem-solving skills at Time 2. This association was not observed from Time 2 to Time 3. To some extent, this interval-specific finding is consistent with concerns regarding the potential negative implications of the authoritarian parenting style in the Chinese cultural context. Authoritarian mothers are typically less emotionally warm and more controlling, and they tend to regulate children’s behavior through verbal hostility or physical punishment (Baumrind, 1967). Such parenting style may limit children’s opportunities to learn constructive strategies for negotiation, perspective-taking, and conflict resolution (Lodeyro et al., 2026). From the perspective of social learning theory, children may acquire behavioral strategies by observing and imitating their mothers’ interaction patterns. When children repeatedly observe that verbal coercion or punishment is used to control others’ behavior, they may be more likely to regard coercive strategies as effective ways to resolve interpersonal conflicts (Lee et al., 2022). As a result, they may be less likely to use positive and reasonable strategies when facing peer conflicts, which may be negatively associated with their social problem-solving skills.

One finding that warrants further discussion is the interval-specific pattern of cross-lagged associations between mothers’ parenting and children’s social problem-solving skills. Specifically, after accounting for children’s prior social problem-solving skills, the authoritative parenting style at Time 1 was not significantly associated with children’s social problem-solving skills at Time 2, whereas the authoritative parenting style at Time 2 was positively associated with children’s social problem-solving skills at Time 3. In contrast, the authoritarian parenting style at Time 1 was negatively associated with children’s social problem-solving skills at Time 2, whereas the corresponding association from authoritarian parenting at Time 2 to children’s social problem-solving skills at Time 3 was non-significant. One tentative, post hoc interpretation concerns differences in children’s social contexts. The Time 1–Time 2 interval included the summer vacation, when children may have experienced fewer kindergarten-based peer interactions and more parent–child contact. The Time 2–Time 3 interval covered a more continuous period of kindergarten life, potentially providing more opportunities for peer interaction, teacher guidance, and conflict-resolution practice (J. Chen et al., 2020). Such contextual differences may help account for the observed pattern. However, parent–child contact, peer exposure, teacher guidance, and children’s imitation of parental behavior were not directly measured. These explanations, therefore, remain speculative, and future research should directly assess the relevant contextual mechanisms to further examine the observed interval-specific associations.

Furthermore, the overall longitudinal associations of fathers’ authoritative and authoritarian parenting with preschool children’s subsequent social problem-solving skills were not significant in the full sample. Previous studies have suggested that mothers’ parenting may be particularly relevant to certain aspects of children’s development (Day & Padilla-Walker, 2009; Desjardins & Leadbeater, 2011). One possible explanation is that, during the preschool years, mothers often serve as children’s primary caregivers and have more frequent daily interactions with them, whereas fathers’ involvement in childcare may be relatively lower (Fivush et al., 2000). In traditional Chinese families, the gendered division of family roles has long been characterized by the notion that “men are responsible for external affairs, whereas women are responsible for domestic affairs”. Although increasing numbers of fathers have taken on childcare and family responsibilities in recent years, mothers still tend to provide more daily care for children under the influence of traditional gender-role expectations (X. Li, 2020). According to the dominance hypothesis, mothers often serve as the central figures in family activities and children’s daily care (Suess et al., 1992). More frequent mother–child interactions may provide mothers with greater opportunities to influence children’s psychological and social development. Thus, no significant longitudinal associations between fathers’ parenting and preschool children’s social problem-solving skills were detected in the full sample.

### 4.2. The Moderating Role of Fathers’ Primary-Caregiver Status

To further clarify whether the longitudinal associations between parenting styles and preschool children’s social problem-solving skills varied depending on whether parents were identified as the primary caregivers, the present study examined the moderating role of primary-caregiver status.

First, primary caregiver status did not significantly moderate the longitudinal associations between mothers’ parenting and children’s social problem-solving skills. One possible explanation is that mothers often establish close emotional bonds with children early in life. Keller (2018) noted that attachment is a special emotional connection that infants develop with significant caregivers during the first year of life. Because early caregiving tasks, such as feeding and daily physical care, are often primarily undertaken by biological mothers, mothers may become central attachment figures for many children. The mother–child attachment relationship may support children’s early regulation abilities and social learning. Moreover, previous research has suggested that mother–child attachment is often stronger than father–child attachment (Kwon & Elicker, 2012). Therefore, even when mothers’ caregiving time decreases, the early attachment relationship and mothers’ parenting styles may remain associated with children’s social development. However, because early caregiving experiences, the amount and quality of parental involvement, and mother–child and father–child attachment were not directly assessed, this attachment-based explanation should be regarded as tentative rather than as an established mechanism and should be examined directly in future research.

Second, an exploratory interaction signal involving fathers’ authoritarian parenting and primary-caregiver status was observed during the Time 1–Time 2 interval. Although the individual interaction coefficient was statistically significant, the joint likelihood-ratio test was non-significant, and the negative simple slope among father-primary-caregiver families did not reach statistical significance. Given these inconsistent results, the limited statistical power, and the small number of father-primary-caregiver families, this finding should be regarded as preliminary and inconclusive rather than as evidence of confirmed moderation. However, this finding highlights the important role of fathers in children’s development and is consistent with the concept of the “important father” proposed by Lamb and Lewis (2013) to some extent. When fathers serve as primary caregivers, they spend more time accompanying and caring for children, which increases opportunities for father–child interaction. According to social capital theory (Shen et al., 2021), parent–child interaction time represents an important form of family social capital and may strengthen the influence of parenting styles on children’s socialization. This finding is broadly consistent with a meta-analysis conducted in China (Ma et al., 2023), which found that parent gender moderated the association between negative parenting and children’s emotional competence, with fathers’ negative parenting showing a stronger adverse effect. In the Chinese cultural context, fathers are often associated with authority and discipline. Chinese fathers were traditionally expected to be emotionally distant educators, disciplinarians, and household heads, even though contemporary fathers are becoming warmer and more involved (X. Li, 2020). Recent qualitative work similarly describes a national setting in which the father’s disciplinarian and authoritarian role remains centralized despite increased caregiving involvement (Wang & Keizer, 2024). However, when fathers interact with children in a highly authoritarian, controlling, or emotionally distant manner, this may increase children’s emotional difficulties, such as anxiety and depression (Carbone et al., 2024). These processes may help explain why fathers’ authoritarian parenting, when fathers are primary caregivers, is not conducive to children’s social problem-solving skills. However, because the frequency and quality of father–child interactions were not directly measured, this explanation remains tentative and requires further investigation.

### 4.3. Limitations and Suggestions for Further Research

This study has several limitations that should be addressed in future research. First, the sample size of the longitudinal study was relatively small, all participants were recruited from Beijing, and the follow-up period lasted only six months. In addition, the attrition rate from Time 1 to Time 3 was 32.54%. Although attrition analyses revealed no significant baseline differences between retained and non-retained participants in the measured demographic and substantive variables, selective attrition cannot be completely excluded. The limited sample size, short follow-up period, and high attrition rate may restrict the generalizability of the findings and the assessment of longer-term developmental processes. Future studies should include families from more diverse regions, including central and western China, as well as rural areas. Extending the tracking interval and duration would also allow researchers to examine more comprehensively the developmental characteristics of preschool children’s social-emotional competence and the mechanisms through which parenting styles influence them.

Second, primary caregiver status was assessed only at baseline and was based mainly on relative caregiving time. Consequently, the measure could not capture possible changes in caregiving arrangements across the three waves or fully reflect parents’ division of specific responsibilities and the quality of their involvement. In addition, the relatively small number of father-primary-caregiver families (*n* = 38) may have limited the statistical power and precision of the moderation estimates. Future studies should assess caregiving involvement multidimensionally and repeatedly over time and recruit larger samples with greater representation of father-primary-caregiver families.

Third, children’s social problem-solving skills were assessed using a three-item subscale completed by the primary caregiver, which may not fully capture this complex construct and may be susceptible to common-method variance. Future studies should combine more comprehensive measures with teacher reports, child-based tasks, or observational assessments.

Fourth, this study did not assess several potential confounding factors, such as child temperament, parental mental health, marital conflict, and teacher or peer influences. Moreover, the traditional cross-lagged panel model cannot distinguish stable between-family differences from within-family changes over time, limiting the interpretation of the cross-lagged associations. Therefore, the present findings should be interpreted as longitudinal associations rather than as evidence of causal or within-family effects. Future research should account for relevant confounding factors and employ random-intercept cross-lagged panel models with larger samples and additional waves of data.

## 5. Conclusions

After accounting for prior levels of children’s social problem-solving skills, mothers’ authoritative parenting at Time 2 was positively associated with children’s social problem-solving skills at Time 3, whereas mothers’ authoritarian parenting at Time 1 was negatively associated with these skills at Time 2. Neither association was replicated across both longitudinal intervals. No evidence was found that primary-caregiver status moderated these maternal associations. Fathers’ authoritative parenting showed no significant longitudinal association with children’s social problem-solving skills. An exploratory Time 1–Time 2 interaction signal involving fathers’ authoritarian parenting and primary-caregiver status was observed, although the evidence remains preliminary. This study contributes to a deeper understanding of the distinct roles fathers’ and mothers’ parenting styles may play in children’s social development and highlights the importance of considering caregiver roles in future research.

## Figures and Tables

**Table 1:** Means and standard deviations of parenting styles and gender differences (*N* = 275).

	Mothers *M* (*SD*)	Fathers *M* (*SD*)	*t*	Cohen’s *d*
T1 authoritative parenting style	4.17 (0.54)	4.05 (0.54)	2.58 ***	0.22
T2 authoritative parenting style	4.25 (0.50)	4.10 (0.55)	3.65 ***	0.29
T3 authoritative parenting style	4.32 (0.51)	4.14 (0.55)	4.58 ***	0.34
T1 authoritarian parenting style	1.71 (0.44)	1.68 (0.53)	0.80	0.06
T2 authoritarian parenting style	1.72 (0.51)	1.66 (0.53)	1.38	0.12
T3 authoritarian parenting style	1.67 (0.50)	1.66 (0.53)	0.07	0.03

Notes. T1 = Time 1, T2 = Time 2, T3 = Time 3, *** *p* < 0.001.

**Table 2:** The inter-correlations among study variables (*N* = 275).

	T1 Social Problem Solving Skills	T2 Social Problem Solving Skills	T3 Social Problem Solving Skills
T1 F_authoritative parenting style	0.16 **	0.12	0.17 **
T1 F_authoritarian parenting style	0.06	0.03	−0.02
T1 M_authoritative parenting style	0.37 ***	0.30 ***	0.30 ***
T1 M_authoritarian parenting style	−0.10	−0.16 **	−0.17 **
T2 F_authoritative parenting style	0.14 *	0.13 *	0.17 **
T2 F_authoritarian parenting style	0.00	0.05	0.02
T2 M_authoritative parenting style	0.23 ***	0.30 ***	0.30 ***
T2 M_authoritarian parenting style	−0.03	−0.11	−0.09
T3 F_authoritative parenting style	0.12	0.09	0.15 *
T3 F_authoritarian parenting style	0.01	0.03	−0.03
T3 M_authoritative parenting style	0.25 ***	0.27 ***	0.38 ***
T3 M_authoritarian parenting style	−0.02	−0.09	−0.09
child gender	0.13 *	0.07	0.15 *
child age	0.08	0.01	−0.02
only child status	0.01	0.10	−0.03
family SES	−0.04	−0.03	−0.05

Notes. F = Fathers, M = Mothers, * *p* < 0.05, ** *p* < 0.01, *** *p* < 0.001; Within-construct correlations across time points are not displayed.

**Table 3:** Model fit results for longitudinal measurement invariance tests of parenting styles and preschool children’s social problem solving skills (*N* = 275).

	Model	S-B*χ*^2^	*df*	CFI	TLI	RMSEA [90% CI]	SRMR	△CFI	△RMSEA	△SRMR
F_authoritative parenting style	Configural invariance	9.965	15	1.000	1.000	0.000 [0.000, 0.031]	0.018	—	—	—
	Metric invariance	13.152	19	1.000	1.000	0.000 [0.000, 0.031]	0.042	0.000	0.000	0.024
	Scalar invariance	20.539	25	1.000	1.000	0.000 [0.000, 0.036]	0.045	0.000	0.000	0.003
F_authoritarian parenting style	Configural invariance	27.140	15	0.990	0.977	0.051 [0.011, 0.084]	0.042	—	—	—
	Metric invariance	30.715	19	0.992	0.984	0.043 [0.000, 0.073]	0.047	0.002	−0.008	0.005
	Scalar invariance	49.857	25	0.981	0.972	0.056 [0.030, 0.081]	0.049	−0.011	0.013	0.002
	Partial scalar invariance	35.311	24	0.990	0.985	0.041 [0.000, 0.069]	0.050	−0.002	−0.002	0.003
M_authoritative parenting style	Configural invariance	11.250	15	1.000	1.000	0.000 [0.000, 0.034]	0.036	—	—	—
	Metric invariance	13.075	19	1.000	1.000	0.000 [0.000, 0.026]	0.043	0.000	0.000	0.007
	Scalar invariance	32.216	25	0.996	0.994	0.028 [0.000, 0.059]	0.050	−0.004	0.028	0.007
M_authoritarian parenting style	Configural invariance	20.880	15	1.000	1.000	0.000 [0.000, 0.057]	0.037	—	—	—
	Metric invariance	23.780	19	1.000	1.000	0.000 [0.000, 0.047]	0.037	0.000	0.000	0.000
	Scalar invariance	38.571	25	0.996	0.995	0.027 [0.000, 0.058]	0.041	−0.004	0.027	0.004
Children’s social problem solving skills	Configural invariance	11.310	15	1.000	1.000	0.000 [0.000, 0.043]	0.017	—	—	—
	Metric invariance	14.764	19	1.000	1.000	0.000 [0.000, 0.039]	0.027	0.000	0.000	0.010
	Scalar invariance	34.595	25	0.988	0.983	0.037 [0.000, 0.065]	0.065	−0.012	0.037	0.038
	Partial scalar invariance	30.079	24	0.992	0.989	0.030 [0.000, 0.061]	0.037	−0.008	0.030	0.010

Note. Partial scalar invariance for paternal authoritarian parenting was established by freeing the intercept of Item 3 at Time 3; partial scalar invariance for children’s social problem-solving skills was established by freeing the intercept of Item 2 at Time 1.

**Table 4:** Simple-slope analysis of the association between fathers’ authoritarian parenting at time 1 and children’s social problem-solving skills at time 2 by primary-caregiver status (*N* = 275).

Primary-Caregiver Status	*n*	*B*	*SE*	95% CI	*p*
Mother as primary caregiver	237	0.07	0.05	[−0.039, 0.173]	0.216
Father as primary caregiver	38	−0.18	0.11	[−0.395, 0.027]	0.088

## Data Availability

The data presented in this study are available on request from the corresponding author. The data are not publicly available due to privacy.

## Abbreviations

The following abbreviations are used in this manuscript:

SES	Socioeconomic Status
LMS	Latent Moderated Structural Equations
AIC	Akaike Information Criterion

## Appendix A. Development and Psychometric Properties of the Social Problem Solving Skills Subscale

The three-item social problem solving skills subscale was derived from the broader Preschool Children’s Social and Emotional Competence Questionnaire developed by the research team. The development process began with conceptual analysis, a review of relevant existing instruments, and qualitative research. Focus-group interviews and individual semi-structured interviews were conducted with 47 kindergarten teachers and 23 parents of preschool children. The interview transcripts were coded using thematic analysis, and the resulting themes informed the preliminary questionnaire framework and item pool.

The proposed dimensions and items were subsequently reviewed by experts to evaluate their consistency with the conceptual content and structure of social-emotional competence. This process provided qualitative evidence of content validity. The questionnaire was then refined through two rounds of exploratory factor analysis and evaluated through a large-scale confirmatory factor analysis involving 2282 preschool children from 11 provinces or municipalities in China. The final social problem solving skills subscale comprised three items describing observable responses to peers’ emotional distress and interpersonal conflict, including “After having a conflict with a friend, the child will make friendly gestures, such as giving small gifts”, “When seeing another child feeling sad or crying, the child takes the initiative to comfort them”, and “When encountering unpleasant situations with one’s peers, one can appropriately manage one’s emotions through comforting, negotiation, and diverting attention”.

A single-factor confirmatory factor analysis was conducted separately at each wave. Since a single-factor model with three indicators is just identified (*df* = 0), global fit indices such as CFI, TLI, RMSEA, and SRMR do not provide an informative assessment of model fit. Accordingly, evidence concerning the factor structure is presented primarily through the magnitude and statistical significance of the standardized factor loadings. The standardized factor loadings ranged from 0.572 to 0.735 at Time 1, from 0.592 to 0.800 at Time 2, and from 0.647 to 0.771 at Time 3. All factor loadings were statistically significant (*p* < 0.001). In addition, Cronbach’s α for the three-item subscale was 0.71 at Time 1, 0.76 at Time 2, and 0.75 at Time 3.

## Appendix B. Sensitivity Analyses Controlling for the Alternative Parenting Dimension

To examine the robustness of the primary findings and address potential omitted-variable bias arising from modeling authoritative and authoritarian parenting separately, two sensitivity models were estimated. In the authoritative-parenting sensitivity model, mothers’ and fathers’ authoritarian parenting measured at the corresponding preceding wave were included as additional covariates in the equations for children’s subsequent social problem solving skills. In the authoritarian-parenting sensitivity model, mothers’ and fathers’ authoritative parenting were included in the same manner. All other model specifications, including the autoregressive and cross-lagged paths, covariates, and within-wave correlations, were kept consistent with the primary models. The authoritative-parenting sensitivity model yielded S-B*χ*^2^ = 475.822, *df* = 325, CFI = 0.954, TLI = 0.944, RMSEA = 0.042, 95% confidence interval [0.034, 0.050], SRMR = 0.094. The authoritarian-parenting sensitivity model yielded S-B*χ*^2^ = 598.897, *df* = 325, CFI = 0.913, TLI = 0.893, RMSEA = 0.061, 95% confidence interval [0.053, 0.069], SRMR = 0.113. The standardized path coefficients, standard errors, 95% confidence intervals, and exact *p* values are reported in Table A1.

**Table A1:** Focal longitudinal path estimates.

Sensitivity Model	Focal Longitudinal Path	*β*	*SE*	95% CI	*p*
Authoritative parenting controlling for authoritarian parenting	T1 M_authoritative parenting style → Social problem solving skills T2	−0.02	0.06	[−0.139, 0.092]	0.691
	T1 F_authoritative parenting style → Social problem solving skills T2	0.01	0.05	[−0.089, 0.104]	0.880
	T2 M_authoritative parenting style → Social problem solving skills T3	0.15	0.05	[0.046, 0.259]	0.005
	T2 F_authoritative parenting style → Social problem solving skills T3	0.07	0.06	[−0.047, 0.181]	0.250
Authoritarian parenting controlling for authoritative parenting	T1 M_authoritarian parenting style → Social problem solving skills T2	−0.12	0.06	[−0.233, 0.000]	0.050
	T1 F_authoritarian parenting style → Social problem solving skills T2	0.04	0.06	[−0.076, 0.148]	0.534
	T2 M_authoritarian parenting style → Social problem solving skills T3	−0.02	0.06	[−0.147, 0.140]	0.714
	T2 F_authoritarian parenting style → Social problem solving skills T3	0.03	0.06	[−0.083, 0.140]	0.620

Note. *β* = standardized coefficient; *SE* = standard error; CI = confidence interval.
